# Interplay Between Sialic Acids, Siglec-E, and Neu1 Regulates MyD88- and TRIF-Dependent Pathways for TLR4-Activation During *Leishmania donovani* Infection

**DOI:** 10.3389/fimmu.2021.626110

**Published:** 2021-03-03

**Authors:** Joyshree Karmakar, Chitra Mandal

**Affiliations:** Cancer Biology & Inflammatory Disorder Division, CSIR-Indian Institute of Chemical Biology, Kolkata, India

**Keywords:** cytokines, MyD88, Neu1, sialic acids, siglec-E, TLR4, TRIF

## Abstract

TLR4 activates two distinct signaling pathways involving adaptors MyD88 and TRIF to produce proinflammatory cytokines and type-I interferon respectively. How *Leishmania donovani* suppresses these pathways is not well studied. We earlier reported, TLR4 is hypersialylated due to reduced membrane-bound neuraminidase (Neu1) on infected-macrophages. We hypothesized that such enhanced sialoglycoconjugates on host cells may modulate the interactions with siglecs- which are the inhibitory receptors. Here, we examined the impact of such sialylation on overall TLR4 activation both in murine cell line J774A.1 and primary bone marrow derived macrophages (BMDM). Supporting this hypothesis, we demonstrated siglec-E engages hypersialylated TLR4 during infection. Such sialic acids-siglec-E interaction enhanced siglec-E phosphorylation that mediated its strong association with SHP1/SHP2 and also upregulated their phosphorylation in both types of macrophages. Pre-treatment of parasites and host cells with neuraminidase reduced SHP1/SHP2 phosphorylation and triggered TLR4 activation respectively through enhanced nuclear translocation of p-65. Moreover, a reciprocal interplay between Neu1 and siglec-E differentially regulates MyD88- and TRIF-pathways through sialic acids on TLR4 as their common substrate during infection. Correspondingly, Neu1 overexpression enhanced MyD88-signaling while still suppressing TRIF-activation. However, silencing siglec-E specifically activated TRIF-signaling. Pro-inflammatory cytokines corresponding to MyD88 and TRIF pathways were also upregulated respectively. Additionally, Neu1 overexpression or siglec-E silencing prevented TLR4 ubiquitination and subsequent degradation by Triad3A. Neu1-overexpression and siglec-E-silencing together followed by infection activated both MyD88 and TRIF-signaling through their enhanced TLR4-association. This elevated the MyD88-specific cytokines and TRIF-mediated IRF3 and IFN-β genes, thus upregulating the pro-inflammatory cytokines and nitric oxide levels and reduced anti-inflammatory cytokines. All these significantly inhibited parasite survival in macrophages thus demonstrating a previously unidentified dualistic regulation of TLR4signaling pathways activation through sialic acids by interplay of Neu1 and siglec-E during Leishmania infection.

## Introduction

Leishmaniasis is a protozoan parasitic disease that manifests in three different forms-cutaneous, mucocutaneous and life-threatening visceral leishmaniasis (VL). VL, caused by *Leishmania donovani* and *Leishmania infantum* is prevalent worldwide and endemic in 62 countries. 90% of cases are reported from Brazil, Bangladesh, India, Nepal and Sudan (1, 2). VL is a deadly neglected tropical disease ranking second to malaria in causing deaths by a protozoal pathogen (3).

Leishmania is intra-macrophage parasite whose initial interaction with the macrophages through the pattern recognition receptors (PRRs) play a vital role in pathogen recognition (4, 5). The toll-like receptors (TLRs) is a well-known PRR that identifies pathogen-associated molecular patterns (PAMPs) and serves as important regulators of the innate immune response (6, 7). Therefore, the initial ability of the parasite to gain entry and ensure its survival relies on the interaction between cell surface molecules and those on the parasite.

However, leishmaniasis is characterized by a defective immune response generally associated with the TLRs of which TLR2, TLR3, TLR4, TLR7, and TLR9 are mainly involved in the disease pathogenesis (8). Among these, TLR4 plays a significant role during VL-infection as its signaling is generally impaired leading to immune suppression (9). AlsoTLR4 is the only known TLR that initiates signaling activation through both myeloid differentiation primary response 88-(MyD88) and Toll/IL-1R domain-containing adaptor-inducing IFNβ-(TRIF)-dependent pathways upon LPS-stimulation (10, 11). LPS-mediated MyD88-signaling in macrophages leads to NF-κB activation and induction of proinflammatory mediators such as TNF-α, IL-1β, and IL-6 (12). Contrary to this, TRIF-signaling activates IFN-regulatory factor 3 (IRF3) which leads to the production of IFN-β, and other IRF3-dependent genes, which ultimately leads to delayed NF-κB activation (11, 13).

Nevertheless, *Leishmania donovani-*infection subverts both these pathways of TLR4 activation (9, 14). We have recently reported MyD88-mediated TLR4 signaling during *L.donovani* infection is impaired and the sialic acids present on TLR4 are an important player for such impairment (15).

Sialic acids are nine carbon keto sugars on the cell surface of higher organisms that mediate cell-cell interactions and play an important role in immune regulation (16–18). Neu1, a sialic acid removing enzyme, is an important regulator of TLR4 activation in LPS-stimulated macrophages (19). So far, only TLR4 is known to be sialylated (20). Neu1 desialylated TLR4 and desialylated -TLR4 dimerized and activated mainly through the MyD88-pathway in LPS-stimulated macrophages (20). Therefore, Neu1-inhibition in LPS-treated macrophages and dendritic cells prevented such MyD88-dependent TLR4-activation (19, 21). Additionally, we reported reduced Neu1 during *L. donovani*-infection enhanced sialylation on TLR4 preventing its association with MyD88 resulting in an inappropriate cellular activation (15). Thus sialic acids on TLR4 are crucial for MyD88-mediated TLR4-signaling.

Sialic acids are also recognized by sialic acid-binding immunoglobulin-like lectins commonly known as siglecs. These membrane receptors have cytoplasmic immunoreceptor tyrosine-based inhibitory motifs (ITIM) (22, 23) that recruit Src homology 2domain containing phosphatase (SHP1/SHP2) which initiate inhibitory signaling (24). These siglecs, mainly siglec 9, are also involved in the regulation of TLR4 activation in LPS-stimulated THP1 cells (21).

Furthermore, modulation of murine siglec-E, an orthologue of human siglec-9, altered TRIF-dependent TLR4 signaling in *E.coli*-infected macrophages and dendritic cells (25). Siglec-E can also bind to sialylated pathogens such as Group B streptococcus (26), *Pseudomonas aeruginosa* (27–29), *L.donovani* (30) and *Trypanosome cruzii* (31) to subvert the host immune response.

All these independent reports suggest that there may be differential activation of MyD88- and TRIF-dependent TLR4 pathways controlled by Neu1 and siglec-E respectively. Whether such regulation during *L.donovani* is determined by Neu1 or siglec-E has not been investigated. Accordingly, here, we attempted to address whether MyD88- and TRIF-mediated TLR4-signaling is dependent either on Neu1 or Siglec-E or both together through their action on the sialic acids of TLR4 during this infection.

Here, we report for the first time that impairment of both Myd88- and TRIF-dependent pathways of TLR4 activation during *L.donovani* infection is accomplished by downregulation of Neu1 thus preventing TLR4 desialylation as well as promoting enhanced TLR4-Siglec-E association to initiate inhibitory signaling. The interplay between Neu1 and siglec-E during *L.donovani* infection modulates the MyD88 and TRIF pathways of TLR4 activation by acting on the TLR4 sialic acids and thus impairing the innate immune arm.

## Materials and Methods

### Chemicals

Bovine serum albumin (BSA), anti-phosphotyrosine (cat no-4G10), anti-phospho-IRF3 (cat no-SAB4504031) and anti-IRF3 (cat no-SAB3500280) antibodies were from Sigma (St.Louis, MO). Neuraminidase from *Arthrobacter ureafacians* (10269611001) was obtained from Roche (Germany). RNeasy Mini Kit was from Qiagen (Limburg, Netherlands); Reverse Transcriptase Kit was from Promega (USA). DyNAmo Color Flash SYBR Green qPCR kit was procured from Thermo Scientific (Rockford, IL). Antibodies-Neu1 (cat no PA5-42552) and Triad3A (cat no-PA5-20079) was from Thermo Fisher Scientific (Rockford, USA). Anti-TLR4 (cat no- MTS510), anti-siglec-E (cat no-377477) antibodies and siglec-E si RNA (cat no-sc-153462) was from Santa Cruz Biotechnology. Anti-Myd88 (cat no-NBP2-27369) and anti-TRIF (cat no-NB120-13810SS) was from Novus Biologicals (Littleton, USA). The cytokine ELISA kits (IFN-β, TNF-α and IL-6) were from Elabscience and from BD-Biosciences (IFN-γ and IL-12). Neu1 plasmid DNA was obtained from Origene (MR226903). All other antibodies were from Cell signaling Technologies (Danvers, MA) unless indicated otherwise. Giemsa stain was obtained from Himedia (India).

### Ethics Statement

The study was performed strictly according to the guidelines framed by the Committee for Control and Supervision of Experiments on Animals (CPCSEA, New Delhi). The protocol was approved by the Institutional Animal Ethics Committee (IAEC) of CSIR-Indian Institute of Chemical Biology (IICB), Kolkata, India with license number 147/1999/CPCSEA. Hamsters and Balb/c mice were maintained in the Animal house of CSIR-IICB under standard climate-controlled (23 ± 2°C; relative humidity 60%) and photoperiod-controlled (12 h light-dark cycles) condition. They were fed with standard rodent pellets supplemented with grain and drinking water ad libitum.

### Parasite Culture

Promastigotes from an Indian *Leishmania donovani (L.d)* strain AG83 (MHOM/IN/1983/AG83) were cultured in M-199 medium containing HEPES buffer (20mM, pH 7.5) supplemented with 10% heat-inactivated fetal calf serum (FCS, v/v), and gentamycin sulfate (200μg/ml) at 22°C. For maintaining the virulence, lag phase promastigotes (2 × 10^7^ cells/100μl) were regularly passaged in the golden hamster. These animals were sacrificed after 4–6 weeks and spleen tissue were collected in sterile phosphate buffer saline (PBS) to obtain amastigotes as described earlier (32).

### Cell Culture

Murine macrophage cell line (J774A.1) obtained from ATCC was cultured in Iscov’s modified Dulbeccos Medium (IMDM) supplemented with 10% heat-inactivated FCS and antibiotic-antimycotic solutions (complete medium) at 37°C with 5% CO_2_. Confluency was maintained by subculturing the cells every 3–4 days as described previously (32).

### Preparation of Bone Marrow Derived Macrophages (BMDM) From Murine Bone Marrow

Bone marrow cells isolated from femurs of BALB/c mice were cultured in RPMI medium supplemented with 10% FCS and 20 ng/ml mouse recombinant M-CSF for 7 days. Thereafter these bone marrow derived macrophages (BMDM) were used as primary macrophages and used for further experiments.

### Neuraminidase Treatment of Macrophages and *L.donovani* Promastigotes

Macrophages (1x10^6^) were either untreated or treated with *Arthrobacter ureafacians* sialidase (10 mU/ml) in serum free medium and incubated for 30 min at 37°C to remove sialic acids from surface. Thereafter, cells were washed with IMDM medium only and infected with *L.donovani* promastigotes (1:10, macrophage: parasites).

Additionally, promastigotes grown in FCS-containing medium were designated as *L.d* ^+sia^ and those treated for 30 min with *Arthrobacter ureafacians* sialidase (10 mU/ml) for removal of sialic acids from parasite cell surface were termed *L.d* ^-sia^. These cells were washed and allowed to infect the macrophages for 30 min at 37°C. Subsequently, these cells were lysed and processed for western blot as described in the following paragraph.

### Pre-Treatment of Macrophages with *Maackia amurensis* lectinII (MALII)

MALII specifically binds to α2,3-linked sialyl residues on cells. Macrophages (1x10^6^) were pretreated with MALII (10 µg/ml) for 30 min (20), washed and infected with *L.donovani* promastigotes for 45 min or left untreated. Subsequently, the cells were processed for lysis and western blotting as detailed in the following paragraph.

### Western Blot and Immunoprecipitation Analysis

Uninfected/infected/transfected and infected J774.A1 and BMDM cells (2 × 10^6^) were washed in PBS and resuspended in lysis buffer containing (150 mM NaCl, 0.1% Triton X-100, 50 mM Tris-HCl pH 8.0) supplemented with protease inhibitors (pepstatin A (1.0μg/ml), aprotinin (10 μg/ml), and 10 μg/ml leupeptin).

Cells were lysed by sonication (Qsonica-LLC, XL-2000 series, Newtown, CT, USA) and centrifuged at 800 × g for 10min. The obtained cell supernatant was further centrifuged at 1,00,000 × g for 30 min for separation of membrane fraction.

The Bicinchoninic acid assay (BCA) was used for protein estimation using bovine serum albumin (BSA) as standard. An equal amount of protein from each sample was separated in SDS-PAGE (10%) under reducing conditions, transferred onto a PVDF, blocked with TBS -2% BSA and incubated with specific primary antibodies of Neu1, siglec-E, p-Tyrosine, p-65,p-IRF3, IRF-3 (1:1,000 dilutions) overnight at 4°C. The blots were subsequently incubated with respective species-specific HRP-conjugated secondary antibody (1:2,000 dilutions) and were developed by West pico enhanced chemiluminescent substrate (ECL) system (Pierce, Thermo Scientific, USA) as described earlier (33).

For immunoprecipitation experiments, cell lysates (500 μg/lane and 150 μg/lane for BMDM), were incubated with specific antibodies (1:100) overnight at 4°C. The antibody-bound complex was pulled down with Sepharose-4B-protein-A.To remove unbound proteins beads were washed with chilled PBS. The bead bound protein complexes were solublized in sample buffer (0.5M Tris pH 6.8, glycerol, 10%SDS) for nonreducing and with β - mercaptoethnol for reducing conditions and separated by SDS PAGE. The blots were developed as stated above. Furthermore, to check for protein loading, the membranes were stripped off with stripping buffer (100 mM β-mercaptoethanol, 2% SDS, 62.5 mM Tris-HCl pH 6.8 for 30 min at 50°C) and reprobed with the same antibodies used for pull down.

Additionally, cells were suspended in a cytosol isolation buffer (10 mM Tris-Cl, 10 mM NaCl, 1.5 mM MgCl_2_, 1 mM PMSF, 0.05% NP-40, pH 6.8), vortexed and centrifuged at 900× g for 5 min at 4°C. This precipitated the nuclear fraction and the supernatant containing the cytosolic fraction was collected. Simultaneously, the pellet was washed with chilled PBS and suspended in nuclear isolation buffer (20 mM Tris-Cl, 137 mM NaCl, 1 mM CalCl_2_, 1 mM MgCl_2_, 1 mM PMSF, 1% NP-40, pH 8.0) for 30 min in ice (29). The suspension was centrifuged at 900× g for 5 min at 4°C and the sup was collected as the nuclear fraction. Both these fractions were similarly processed for western blot analysis. The band intensities for each band were determined using ImageJ software and represented as bar diagram.

### Confocal Microscopy

Uninfected or infected macrophages (2 × 10^4^) were allowed to adhere on the coverslips. Thereafter, cells were washed with PBS and fixed in paraformaldehyde (4%) for 15 min. The cells were incubated with rabbit anti-TLR4 and mouse anti-siglec-E antibodies followed by staining with rabbit Alexafluor 488 and mouse alexafluor 647 respectively. They were mounted in a media containing DAPI to stain the nucleus and examined on an inverted confocal microscope.

### Transfection

Neu1-plasmid DNA or Siglec-E small interfering RNA (siRNA) were transiently transfected into J774.A1 and BMDM (1 × 10^6^/well) in transfection medium- Opti-MEM using the lipofectamine and Plus reagent (Invitrogen) according to the manufacturer’s instructions (34). After 6 h the transfection medium was replenished with fresh medium. Mock-transfected cells were used as the control for all the experiments. Thereafter, cells were infected with stationary phase promastigotes for 2 h. Overexpression of Neu1 or silencing of siglec-E was verified by western blotting.

### Genetic Expression Profiling by Real-Time PCR

Untransfected, Neu1-transfected and siRNA-siglec-E-transfected J774A.1 cells (1 × 10^6^/well) were infected with the stationary phase promastigotes of *L. donovani* at a ratio 1:10 for 4 h in a six-well plate. Unbound parasites were washed out and infection was allowed for an additional 20 h.

Total RNA from untransfected, untransfected-infected, Neu1-overexpressed and siglec-E-silenced -*L.d* infected J774A.1 cells were extracted using the RNeasy mini kit following the manufacturer’s instruction. First-strand cDNA was synthesized from template RNA (1 μg) by ImPromII-Reverse transcription system. Real-time PCR was performed with specific mice primers for IL-1β, TNF-α, IL-6, IFN-β, and pro-inflammatory (IL-12 and IFNγ), anti-inflammatory cytokines (IL-10 and TGF-β), and iNOS (**Table 1**) obtained from Eurofins Genomics India Pvt. Ltd. using a DyNAmo Flash SYBR Green qPCR Kit. Light Cycler 96 (Roche) software was used to quantitate relative amounts of target mRNA with 18S rRNA as an internal control. Data were expressed as a fold change compared with uninfected control using the comparative cycle threshold (CT) method (35).

**Table 1:** List of primers and their sequences (forward and reverse) used for quantitative real time-PCR.

Primer		Primer sequence(5’-3’)	Tm(°C)
IL-1β	Forward	CCATTAGACAACTGCACTAC	50
	Reverse	CACAGGACAGGTATAGATTC	50
TNF-α	Forward	GCACCACTAGTTGGTTGTCT	52
	Reverse	GACCCTCACACTCAGATCAT	52
IL-6	Forward	AATGATGGATGCTACCAAAC	48
	Reverse	TAGCCACTCCTTCTGTGACT	52
IFN-β	Forward	GAGTTCATCCAGGAGACGTA	50
	Reverse	CAAGATCCCTATGGAGATGA	50
IL-12	Forward	CAGGATGGAGAATTACAGGA	51.2
	Reverse	GTTATTGAGGGCTTGTTGAG	51.2
IFNγ	Forward	CAGGTGGCATAGATGTGGAAGA	54.7
	Reverse	GTGGGTTGTTGACCTCAAACTT	55.7
iNOS	Forward	ACCTGAAAGAGGAAAAGGAC	52.2
	Reverse	GGAGCCATAATACTGGTTGA	51.7
IL-10	Forward	CTAACGGAAACAACTCCTTG	51.0
	Reverse	GAAAGGACACCATAGCAAAG	51.2
TGF-β	Forward	CCCTAGATTTTGACTTGCAC	51.2
	Reverse	GCCCAGTCACTAAGACTCTG	54.7
18S rRNA	Forward	GCTCATTAAATCAGTTATGG	46.0
	Reverse	ACTACCATCGAAAGTTGATA	46.0

### Determination of Secreted Cytokines and Nitric Oxide (NO)

Untransfected, Neu1-transfected and siRNA-siglec-E-transfected cells were infected and processed as mentioned above. Cell-free culture supernatant was collected and assessed for the accumulated cytokines using a sandwich ELISA kit following the manufacturer’s protocol and nitrite was estimated using Griess assay (36).

### Measurement of Intracellular Parasites by Giemsa Staining

Resident peritoneal macrophages were obtained from BALB/c mice (8–10 weeks old) by injecting 5–10ml chilled PBS supplemented with 3% FCS into the peritoneal cavity. The peritoneal exudates cells were pulled out using a syringe, centrifuged (200 × g, 5 min), and washed with PBS. These cells (5 × 10^5^ cells) were grown on 22 mm glass coverslip in complete medium for 20 h for adherence and transfected followed by infection with stationary phase promastigote. Thereafter, cells were washed with medium after 4hr to remove unbound cells. Infection was allowed for an additional 20 h. Cells were subsequently fixed in methanol and stained with Giemsa. The amastigotes were counted manually under a light microscope (EVOS, Life Technologies) at oil immersion (100x) (15, 32).

### Statistical Analysis

All the data were the mean value from three independent experiments and statistical analysis was performed using Graph Pad Prism5. Standard error bars represent the standard deviation of the mean (± S.D.) and statistically significant differences between experimental groups were calculated by Student’s t test. *p ≤ 0.05, **p ≤ 0.01 and ***p ≤ 0.001 were considered significant.

## Results

### Enhanced Association of Siglec-E with TLR4 Modulated ITIM Signaling

**Figure 1:**
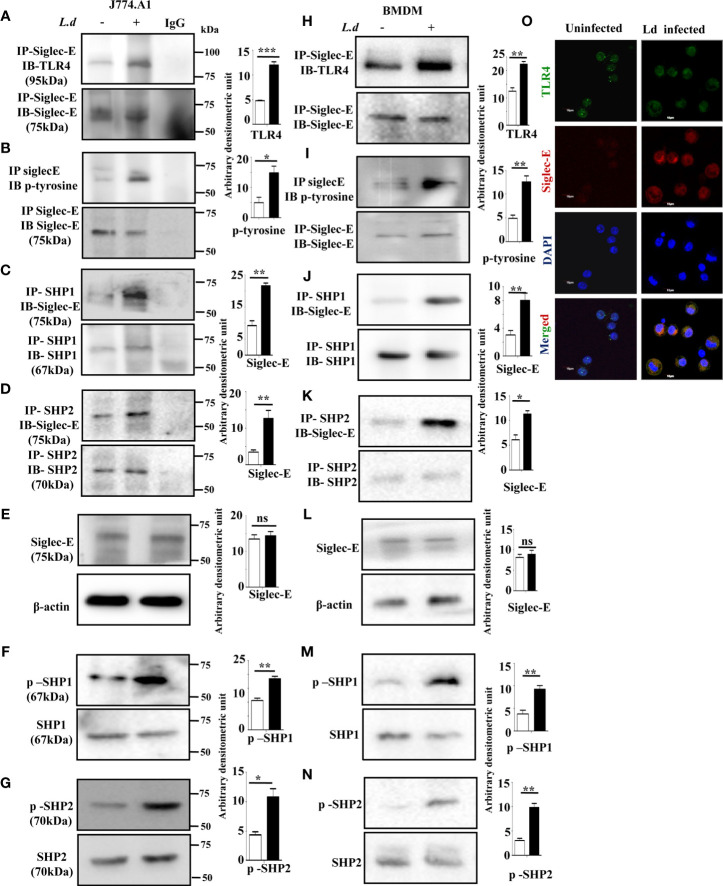
Enhanced association of siglec-E with TLR4 modulates immunoreceptor tyrosine-based inhibitory motifs (ITIM) signaling during *L.d* infection. **(A–D)** J774.A1 cells (1x10^6^/well) were either left uninfected or infected with stationary phase *L.d* promastigotes in a six-well plate at a 1:10 ratio for 2 h. The cell lysates obtained from these cells were incubated with **(A, B)** anti-siglec-E, **(C)** anti-SHP1, and **(D)** anti-SHP-2 antibodies for overnight and immunoprecipitated with protein A beads and processed as mentioned in material and methods. The blots were subsequently probed with anti-TLR4 **(A)** anti-p-Tyrosine **(B)** or anti-siglec-E **(C, D)** antibodies. These blots **(A–D)** were also stripped and reprobed with the respective antibodies incubated with for immunoprecipitation to show equal loading. The IgG lane served as negative control. Additionally, blots were also incubated with **(E)** anti-siglec-E, **(F)** anti-p-SHP1, anti-SHP1, **(G)** anti-p-SHP2, anti-SHP2 and β-actin (loading control). **(H–K)** As mentioned for J774.A1 cells above, bone marrow derived macrophages (BMDM) were also similarly treated and the blots were probed with anti-TLR4 **(H)** anti-p-Tyrosine **(I)** or anti-siglec-E **(J, K)**. **(L–N)** Blots were also incubated with anti-siglec-E **(L)**, anti-p-SHP1, anti-SHP1 **(M)**, anti-p-SHP2, anti-SHP2 **(N)** and β-actin to show loading control. **(O)** J77.A1 cells (2x10^4^) either uninfected or infected were paraformaldehyde fixed and processed as in materials and methods. Enhanced siglec-E-TLR4 association was visualized in the infected macrophages as indicated by their enhanced colocalization. Scale bar=10µM. The experiments were repeated thrice and representative immunoblots were shown. Bar graphs show the band intensities of the blots measured by ImageJ software. The significance between different experimental groups was calculated by student’s t-test using Graph pad prism 5 software. Significance: *p ≤ 0.05, **p ≤ 0.01, ***p ≤ 0.001, ns-not significant.

Siglec-E is a well-known negative regulator of immune cell activation and it interacts with sialic acids on TLR4. Therefore, we examined whether such association between siglec-E and TLR4 also exists during *L.donovani* infection. Enhanced interaction between siglec-E and TLR4 was observed upon immunoprecipitating siglec-E from cell lysates with anti-siglec-E antibodies and blotting with anti-TLR4 in the infected cells compared to the uninfected counterpart (**Figure 1A**).

Siglec-E is an inhibitory receptor containing ITIM responsible for negative regulation. Binding to sialylated ligands leads to its phosphorylation and subsequent recruitment of phosphatases by the ITIMs. We, therefore, checked the phosphorylation of siglec-E during *L.donovani* infection. Enhanced tyrosine phosphorylation of siglec-E was observed in the infected macrophages compared to uninfected cells (**Figure 1B**) by immunoprecipitation experiments. We next sought to examine if phosphorylated siglec-E could recruit SHP1 and SHP2. A strong association of siglec-E with SHP1 (**Figure 1C**) and SHP2 (**Figure 1D**) was depicted in the infected cells in comparison to the uninfected counterparts by immunoprecipitation assay. However, the protein level of siglec-E was similar in both uninfected and infected cells (**Figure 1E**). This further prompted us to check if the phosphatases SHP1 and SHP2 were phosphorylated which indicates their activation. Enhanced phosphorylated SHP1 and SHP2 were indeed observed in the *L.donovani* infected cells compared to uninfected cells (**Figures 1F, G** respectively). To further, substantiate our findings, all these experiments were also repeated in the primary mouse bone marrow derived macrophages (BMDM) (**Figures 1H–N**). They exhibited a similar trend of enhanced siglec-E-TLR4 association as well as an upregulated siglec-E signaling as demonstrated by the J774.A1 macrophages (**Figures 1A–G**).

Confocal microscopy studies further confirmed our observation of enhanced siglec-E-TLR4 association during the infected condition as displayed by higher siglec-E-TLR4 colocalization in the infected macrophages (**Figure 1O**) compared to uninfected control. DAPI stained the intact macrophage nucleus in blue. These data indicate that *L.donovani* infection modulated the ITIM signaling associated with siglec-E through recruitment of SHP1 and SHP2 and their phosphorylation.

### Sialic Acids on TLR4 Regulates Its Association With Siglec-E

**Figure 2:**
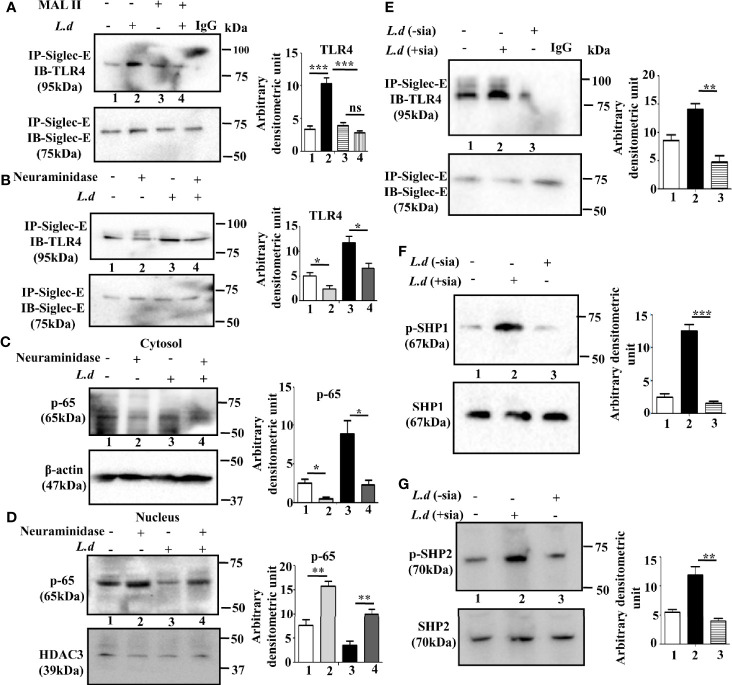
Sialic acids on toll-like receptor 4 (TLR4) regulates its association with siglec-E. **(A, B)** J774.A1 cells were either left uninfected or infected or *Maackia amurensis* lectinII (MALII) treated (uninfected or infected) or neuraminidase treated uninfected and infected with promastigotes as stated in **Figure 1**. The cell lysates were incubated with the anti-siglec-E antibody for overnight and immunoprecipitated with protein A beads. Thereafter, the precipitates were analyzed by SDS-PAGE under non-reducing conditions and subsequently probed with anti-TLR4 antibody **(A, B)**. IgG lane was used as negative control. **(C, D)** Cytosol and nuclear fractions were separated from cell lysates. They were subsequently separated by SDS-PAGE. Status of cytosolic **(C)** and nuclear **(D)** p-65 were determined by western blotting. β-actin and HDAC3 were used as the loading control for cytosol and nucleus respectively. **(E–G)** Additionally, the parasites were also neuraminidase treated as mentioned in materials and methods and then used for infecting macrophages. The cell lysate from this cell was immunoprecipitated with anti-siglec-E antibody and blotted with anti-TLR4 **(E)**. Blots were also developed for anti-p-SHP1, anti-SHP1 **(F)**, and anti-p-SHP2, anti-SHP2 **(G)**. Representative Immunoblots are shown. Band intensities of the blots are represented by bar graphs. Significance: *p ≤ 0.05, **p ≤ 0.01, ***p ≤ 0.001.

TLR4 is a sialylated molecule and interaction of siglecs with their ligands depends on sialic acids. Therefore, to confirm, that siglec-E binds with sialyl residues on TLR4, we pretreated the cells with *Maackia amurensis* lectin II that specifically identifies α2,3-linked sialic acids (**Figure 2A**). In the MALII pre-treated uninfected (lane 3) or *L.donovani* infected (lane 4) cells, the level of siglec-E-TLR4 association was comparable. However, an enhanced association of siglec-E with TLR4 was observed in lectin-untreated infected cells (lane 2). This data indicates the involvement of sialic acids in such interaction during *L.donovani* infection.

To further confirm, the involvement of sialic acids during infection, we investigated the effect of exogenous neuraminidase (*Arthrobacter ureafaciens*) on such interaction. As shown in **Figure 2B**, siglec-E-TLR4 binding was reduced in the neuraminidase treated cells (lane2) compared to untreated cells (lane 1). This trend of reduced siglec-E-TLR4 association was also exhibited when neuraminidase treatment was followed by *L.donovani* infection (lane4) compared to only parasite-infected cells (lane3). This showed that interaction of TLR4 with siglec-E is sialic acid mediated.

To further elucidate that sialyl residues are responsible for the regulation of TLR4-mediated signaling, the nuclear translocation of p-65 was assessed in the infected and uninfected cells with or without neuraminidase treatment. The level of p-65 was elevated in the nucleus compared to the cytosol in the neuraminidase-treated uninfected and infected cells (**Figures 2C, D**). This indicated that removal of sialic acid leads to cellular activation through nuclear translocation of p-65 which was also evident in neuraminidase pre-treated *L.donovani* infected cells. This further confirmed the involvement of sialic acids in siglec-E-TLR4 interaction.

Sialic acids are also abundantly present on the surface of *L.donovani* promastigotes. Therefore, we further examined if promastigote sialic acids has any role in siglec-E-TLR4 association during infection. Such association was reduced in the macrophages infected with neuraminidase treated parasites (*L.d* ^-sia^) (**Figure 2E**, lane 3), compared to those treated with *L.d* ^+sia^ (**Figure 2E**, lane 2). Furthermore, phosphorylation of SHP1 (**Figure 2F**, lane3) and SHP2 (**Figure 2G**, lane3) was also reduced in the cells infected with *L.d* ^-sia^ compared to the *L.d* ^+sia^ infected cells. All these data suggest an important role of sialic acids in TLR4-siglec-E interaction during *L.donovani* infection. This association depends on both host and parasite sialic acids.

### Reduced Siglec-E-ITIM Signaling by Modulation of Neu1 or Siglec-E

So far we have established that exogenous sialidase treatment leads to TLR4 activation by abrogating sialic acid-dependent TLR4-siglec-E interaction in the infected cells. We have earlier reported that Neu1 on leishmania-infected cells is down-regulated leading to enhanced sialylation on TLR4. Furthermore, overexpression of Neu1 activates TLR4 due to the removal of sialic acids (15). Additionally, TLR4 activation is negatively regulated by siglec-E, due to interaction with the sialic acids present on TLR4. We, therefore, checked the relevance of Neu1 and siglec-E on TLR4 activation during parasite infection.

**Figure 3:**
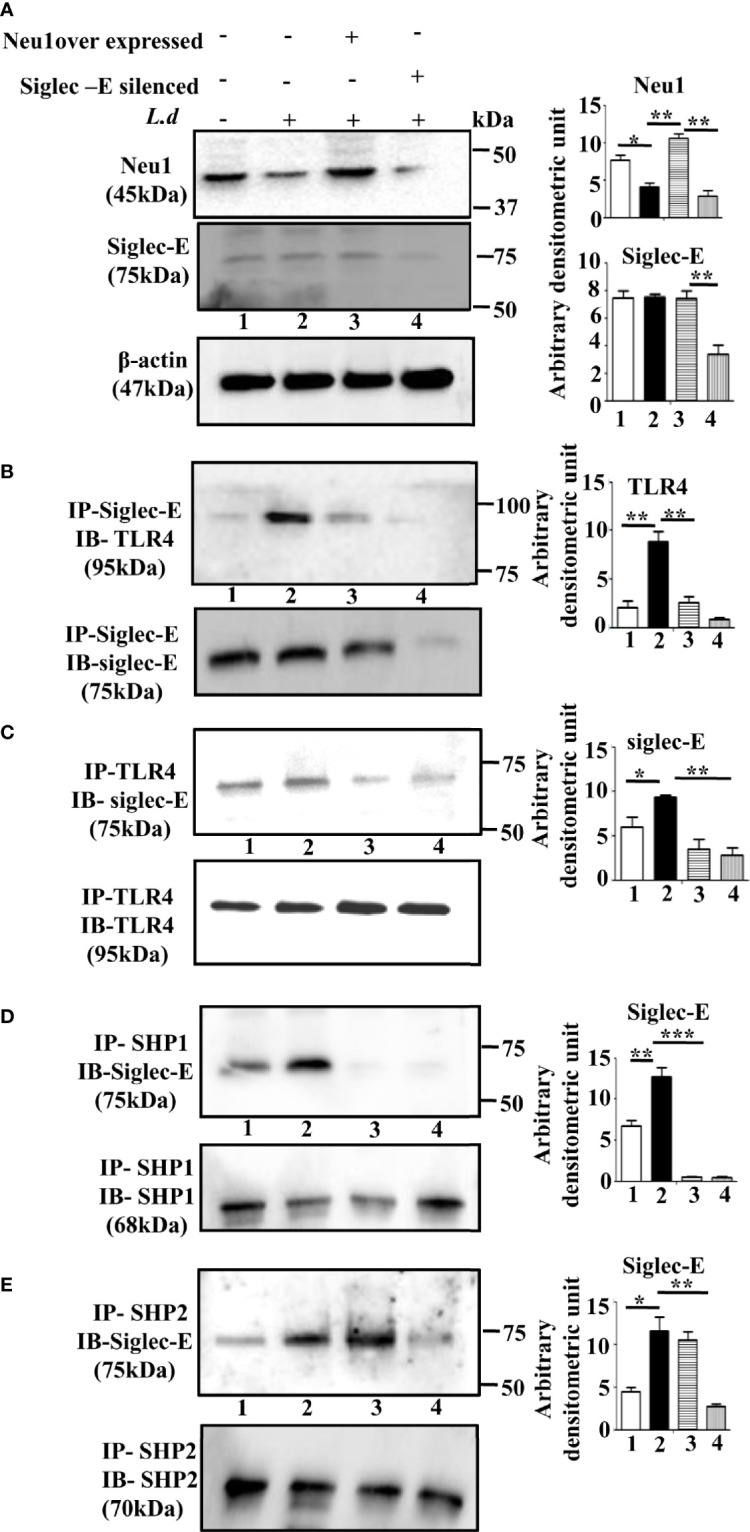
Alteration of siglec-E-toll-like receptor 4 (TLR4) association and siglec-E- immunoreceptor tyrosine-based inhibitory motifs (ITIM) signaling by modulation of Neu1 or siglec-E during this parasite infection. **(A)** J774.A1 cells (1x10^6^/well) were either untransfected or untransfected and infected with stationary phase promastigotes for 2 h or Neu1-transfected and infected or siglec-E si RNA-transfected and infected as described in materials and methods. Cell lysates from these cells were used for western blot analysis to determine the status of Neu1, siglec-E protein. β-actin was used to show equal loading. Representative blots showed enhanced Neu1 expression in Neu1 overexpressed infected cells and reduced siglec-E levels in siglec-E siRNA-transfected -infected cells. **(B–E)** Cell lysates were next immunoprecipitated with **(B)** anti-siglec-E or **(C)** anti-TLR4 or **(D)** anti-SHP1 or **(E)** anti-SHP2 antibodies respectively and proceeded further as described in **Figure 1**. These blots were probed with **(B)** anti-TLR4 or **(C–E)** anti-siglec-E antibodies. These blots were stripped and reprobed with the corresponding IP antibodies. Experiments were repeated thrice and representative blots are shown. Bar graph represents the band intensities. IP, immunoprecipitation; IB, Immunoblot. Significance: *p ≤ 0.05, **p ≤ 0.01, ***p ≤ 0.001.

Accordingly, we overexpressed Neu1, which would desialylate and activate TLR4 and silenced siglec-E to prevent any binding with sialic acids which were followed by infection and confirmed by western blot analysis (**Figure 3A**). β-actin showed equal loading.

Next, we checked the siglec-E-TLR4 association in these situations. We observed reduced TLR4-siglec-E association both in Neu1 overexpressed and siglec-E silenced-infected cells compared to untransfected infected cells when immunoprecipitated with anti-siglec-E antibody and blotted with anti-TLR4 antibody (**Figure 3B**). Furthermore, we also immunoprecipitated with anti-TLR4 and blotted with anti-Siglec-E (**Figure 3C**), which demonstrated similar trend of reduced siglec-E-TLR4 association in the Neu-1 over expressed and siglec-E silenced infected cells.

This encouraged us to explore ITIM-dependent signaling. As expected, under siglec-E silenced condition the association of siglec-E with SHP1 (**Figure 3D**, lane4) and SHP2 (**Figure 3E**, lane 4) was greatly reduced. However, when Neu1 was overexpressed, this association was reduced in the case of SHP1 (**Figure 3D**, lane3), but siglec-E-SHP2 association was not completely reduced and was comparable to untransfected infected cells (**Figure 3E**, lane3). These observations indicate that the desialylation of TLR4 or inhibiting the interaction of sialic acids on TLR4 with siglec-E disrupts not only the siglec-E-TLR4 binding but also ITIM signaling during parasite infection. Furthermore, while siglec-E was solely responsible for activation of ITIM signaling by interacting with sialic acids on TLR4, Neu1 was partly involved in diminishing such binding through desialylation.

### Neu1 Upregulation Activates the MyD88-and Siglec-E Silencing Augments TRIF-Dependent TLR4 Signaling

TLR4 activation, in the early phase, is initiated by the recruitment of adapter MyD88 through the removal of sialic acids present on TLR4. Therefore, either removal of these sialic acids or preventing them from binding to siglec-E may lead to TLR4 activation.

**Figure 4:**
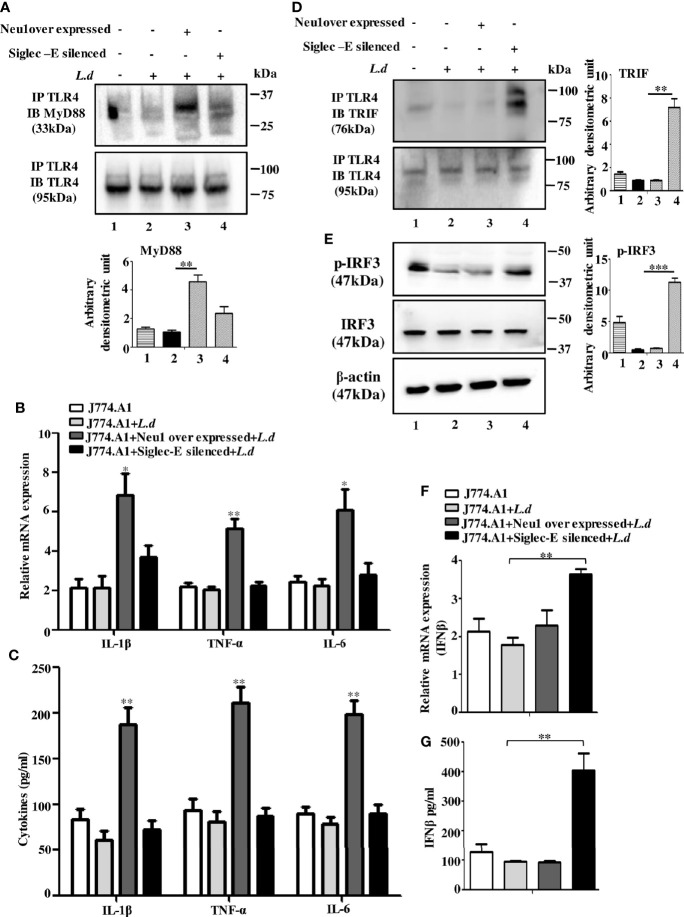
Neu1-overexpression activates MyD88- and siglec-E-silencing augments the TRIF-pathways of toll-like receptor 4 (TLR4)-activation in *L.d* infected macrophages. J774.A1 cells (1x10^6^/well) were transfected and infected as stated in **Figure 3**. **(A)**. The lysates from these cells were immunoprecipitated with anti-TLR4 and subsequently blotted with anti-MyD88. **(B)** Total RNA isolation and cDNA were prepared as described in materials and methods. Relative mRNA expression was determined for IL-1β, TNF-α, and IL-6 using specific primers. **(C)** The levels of the secreted cytokines (IL-1β, TNF-α, and IL-6) were also determined by ELISA. **(D)** Similarly, cell lysates were immunoprecipitated with anti-TLR4 and blotted with anti-TRIF antibodies. **(E)** The lysates were also analyzed for phosphorylated and non-phosphorylated IRF3 by immunoblotting. β-actin was used to show equal loading. Bar graphs represent the band intensities of the blots. **(F, G)** As stated above, the genetic expression of IFN-β **(F)** and its level in secreted cell supernatant **(G)** were checked. The genetic expression was normalized against 18s rRNA. The significance between different experimental groups was calculated by student’s t-test using Graph pad prism 5 software. Significance: *p ≤ 0.05, **p ≤ 0.01, ***p ≤ 0.001.

Accordingly, we examined the association between TLR4 and MyD88 under Neu1 overexpressed or siglec-E silenced cells in uninfected and *L.donovani* infected conditions (**Figure 4A**). As expected the TLR4-MyD88 association was almost undetectable in the *L.donovani* infected cells (lane2) However, this association was significantly enhanced in the Neu1 overexpressed infected cells (lane3). Similarly, the siglec-E-silenced infected cell (lane4) also displayed a reduced level which was comparable to untransfected-infected cells (lane2).

We next, studied the impact of Neu1 overexpression or siglec-E silencing on the expression of MyD88-dependent cytokines. In response to *L.donovani* infection following Neu1 overexpression, there was a significantly greater expression of IL-1β (p ≤ 0.0175, ~ 3 fold), TNF-α (p ≤ 0.0046, ~ 2.5 fold) and IL-6 (p ≤ 0.0242, ~ 2.7fold) when compared to un transfected-infected or siglec-E silenced infected cells (**Figure 4B**). We also checked the level of these cytokines in secretion in cell supernatant. Corroborating with the genetic expression data, we observed significant upregulation of IL-1β (p ≤ 0.0042, ~ 3 fold), TNF-α (p ≤ 0.003, ~ 3 fold)) and IL-6 (p ≤ 0.002, ~ 2.5 fold) fold only in the Neu1 overexpressed condition (**Figure 4C**). This data indicates that only the desialylation of TLR4 by Neu1 leads to activation of the MyD88 pathway.

Since Neu1 and siglec-E both recognize sialic acids on TLR4 as their binding partner, we now explored if they regulated the TLR4-triggered activation of the TRIF pathway. We, therefore, examined the recruitment of TRIF to TLR4 by immunoprecipitation (**Figure 4D**). The association of TRIF with TLR4 was specifically upregulated when siglec-E was silenced (lane4). However, upon Neu1 overexpression, such association remained comparable to infected cells, which was in contrast to the MyD88 pathway shown in **Figure 4A**.

TRIF-activation subsequently leads to the cascade of the TRIF-IRF3-IFNβ signaling axis. To further confirm these observations, we checked the phosphorylation of IRF3 (**Figure 4E**). While significant levels of phosphorylated IRF3 were detected in siglec-E silenced *L.donovani* infected cells (**Figure 4E**, lane4) this was reduced both in untransfected infected (lane2) and Neu1 overexpressed infected (lane3) cells.

Furthermore, siglec-E silenced infected cells exhibited significantly (p ≤ 0.0015) enhanced expression of IFN-β production by ~2 fold compared to un-transfected-infected and Neu1 overexpressed infected cells (**Figure 4F**). This was further corroborated by significantly (p ≤ 0.0068) enhanced production of IFN-β by ~ 4 fold in the cell supernatant (**Figure 4G**). These data indicate that siglec-E is specifically involved in the modulation of TRIF-mediated TLR4 signaling which is dependent on the presence of sialic acids on TLR4 in *L.donovani* infected macrophages. Thus siglec-E prevents TRIF-activation by binding to the sialic acids of TLR4.

### Genetic Modulation of Neu1 or Siglec-E Prevents TLR4 Ubiquitination and Degradation During Parasite Infection

**Figure 5:**
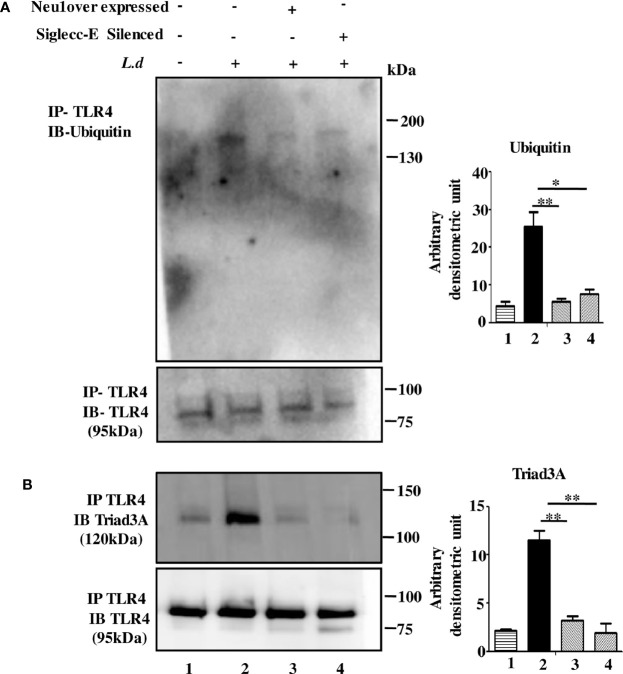
Neu1 overexpression or siglec-E silencing prevented toll-like receptor 4 (TLR4) degradation. **(A, B)** Macrophages (untransfected or transfected) followed by infection were lysed. The lysates were immunoprecipitated with anti-TLR4 and further processed as mentioned in materials and methods. The blots were probed with anti-ubiquitin **(A)** and anti-Triad3a **(B)**. Data were obtained from three independent experiments and one representative blot is shown. Bar graphs represent band intensities. Significance: *p ≤ 0.05, **p ≤ 0.01.

Microbial encounter generally downregulates TLR4 signaling through its ubiquitin-mediated TLR4 degradation. So, we hypothesized that the hypersialylated TLR4 may be degraded leading to signal termination during this parasitic infection. Accordingly, we checked the ubiquitination of TLR4 by immunoprecipitation. We observed enhanced TLR4 ubiquitination in infected cells but this was reduced in Neu1-overexpressed or siglec-E silenced-infected cells (**Figure 5A**).

Such ubiquitination may involve the E3-ubiquitin ligase (Triad3A) leading to degradation of TLRs. While the TLR4-Triad3A association was elevated in the un-transfected infected cells, such interaction was undetected both in Neu1-overexpressed or siglec-E-silenced *L.donovani* infected cells (**Figure 5B**).

Thus our data indicate TLR4 is ubiquitinated and degraded during infection which is inhibited under Neu1 overexpressed or siglec-E silenced infected conditions. This suggests that siglec-E is unable to bind to sialic acids of TLR4 as Neu1 desialylated TLR4. Thus both Neu1 and siglec-E determine the fate of TLR4.

### An Interplay Between Neu1 and Siglec-E in TLR4 Activation During *L.donovani* Infection

So far we have demonstrated that Neu1 is downregulated and the siglec-E-TLR4 association is enhanced leading to improper TLR4 signaling during *L.donovani* infection. There was a differential regulation of TLR4-activation pathways during this infection. One mediated by Neu1 and the other by siglec-E and both are dependent on sialic acid of TLR4.

**Figure 6:**
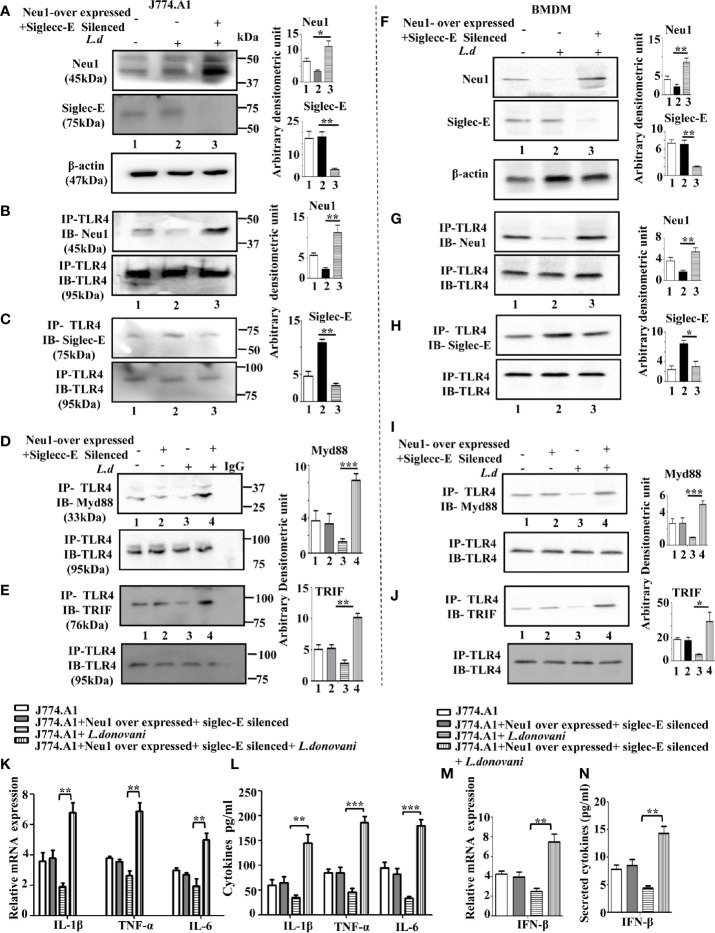
Upregulation of MyD88- and TRIF dependent toll-like receptor 4 (TLR4) activation in Neu1 overexpressed and siglec-E silenced co-transfected-infected cells. **(A)** J774.A1 cells (1x10^6^/well) were untransfected, mock-transfected and Neu1overexpressed along with siglec-E-silenced co-transfected followed by *L.donovani* infection. The lysates from these cells were used to visualize the protein levels of Neu1 and siglec-E **(A)**. β-actin was used for equal loading. **(B–E)** In another set of experiments, the lysates were incubated with anti-toll-like receptor 4 (TLR4) antibody overnight and immunoprecipitated. The blots were probed with anti-Neu1 **(B)**, anti-siglec-E **(C)**, anti-Myd88 **(D)**, and anti-TRIF **(E)** antibodies. Each blot is representative of three independent experiments. **(F)** BMDM (1x10^6^/well) was similarly processed as in **(A)** above. Protein levels of Neu1 and siglec-E were visualized. **(G–J)** Cell lysates from bone marrow derived macrophage (BMDM) cells were similarly treated as mentioned for **(B–E)** above. Associations of TLR4 with **-** Neu1 **(G),** siglec-E **(H),** MyD88 **(I),** and TRIF **(J)** were visualized in cell lysates of primary cells. **(K)** Relative mRNA expression was determined for IL-1β, TNF-α, and IL-6 using specific primers. **(L)** Cell-free culture supernatant from these cells was collected and the level of secreted cytokines- IL-1β, TNF-α, and IL-6 was measured by ELISA. **(M, N)** The genetic expression of IFN-β **(M)** and its level in secreted cell supernatant **(N)** was also determined. Band intensities are represented in the bar graphs. Data represented are mean ± SD of three independent experiments. The significance between the experimental groups was calculated by Student’s t-test. Significance *p ≤ 0.05, **p ≤0.01, ***p ≤0.001.

We, therefore, hypothesized a proper balance of Neu1 and siglec-E is needed for effective activation of TLR4 both by MyD88 and TRIF. Accordingly, we overexpressed Neu1 and silenced siglec-E together by co-transfection and infected with *L.donovani* parasites and checked their status by western blot in J774.A1 macrophages (**Figure 6A**).

Proper TLR4 activation depends on its proximity with either Neu1 or siglec-E. We, therefore, checked the interaction of TLR4 with Neu1 and with siglec-E under co-transfected infected condition. We observed an enhanced TLR4-Neu1 (**Figure 6B**) and reduced TLR4-siglec-E (**Figure 6C**) associations in the co-transfected infected cells compared to mock-transfected infected cells.

Next, we addressed if such co-transfection could activate both MyD88 and TRIF pathways of TLR4 activation. Accordingly, we first checked the association of TLR4 with MyD88 (**Figure 6D**) under the co-transfected conditions followed by *L.donovani* infection. Our data displayed an enhanced association of TLR4 with MyD88 compared to mock-transfected infected cells. We further examined the TLR4-TRIF association in Neu1-overexpressed and siglec-E silenced co-transfected cells followed by infection as measured by co-immunoprecipitation (**Figure 6E**). Our data displayed an enhanced TLR4-TRIF association in the co-transfected-infected condition compared to mock-infected cells.

To substantiate these important findings, we further checked the association of all these molecules in the BMDM (**Figures 6F–J**). We observed an enhanced Neu1 and reduced siglec-E (**Figure 6F**) in the Neu1 overexpressed and siglec-E silenced co-transfected cells ensuring proper transfection in these cells.

As expected, these cells also displayed enhanced TLR4- Neu1 (**Figure 6G**) and reduced TLR4-siglec-E (**Figure 6H**) association in these co-tranfected infected primary cells. Moreover, these cells also demonstrated enhanced TLR4-MyD88 (**Figure 6I**) and upregulated TLR4-TRIF association in the co-transfected cells following infection.

Encouraged by these data, we examined the genetic expression of pro-inflammatory cytokines activated by the TLR4-MyD88 pathway. We observed a significantly enhanced expression of IL-1β (p ≤ 0.0021, ~3.6fold), TNF-α (p ≤ 0.0027, ~2.61fold) and IL-6 (p ≤ 0.0085, ~2.5fold) (**Figure 6K**). Additionally, we checked the production of these cytokines in the cell supernatant which displayed significantly upregulated IL-1β (p ≤ 0.0039, ~4.2 fold), TNF-α (p ≤ 0.0039, ~4.03 fold) and IL-6 (p ≤ 0.0001, ~5.4fold), (**Figure 6L**), which corroborated with the genetic expression data. The co-transfected uninfected cells did not demonstrate any such change. We also checked the expression of signature molecule IFNβ of the TLR4-TRIF pathway at the genetic level, which was significantly (p ≤ 0.0043) upregulated by ~3.1 fold (**Figure 6M**). A significantly (p ≤ 0.0017) enhanced secreted IFN-β in the cell supernatant by 3.32 fold was observed (**Figure 6N**).

Together, these data indicate the sialic acids on TLR4 are a crucial factor for its proper activation during *L.donovani* infection. Also, such interaction relies on the interplay between Neu1 and siglec-E to upregulate both MyD88 and TRIF mediated TLR4 signaling activation.

### Activation of the Antileishmanial Immune Response in the Presence of both Neu1 and Siglec-E

**Figure 7:**
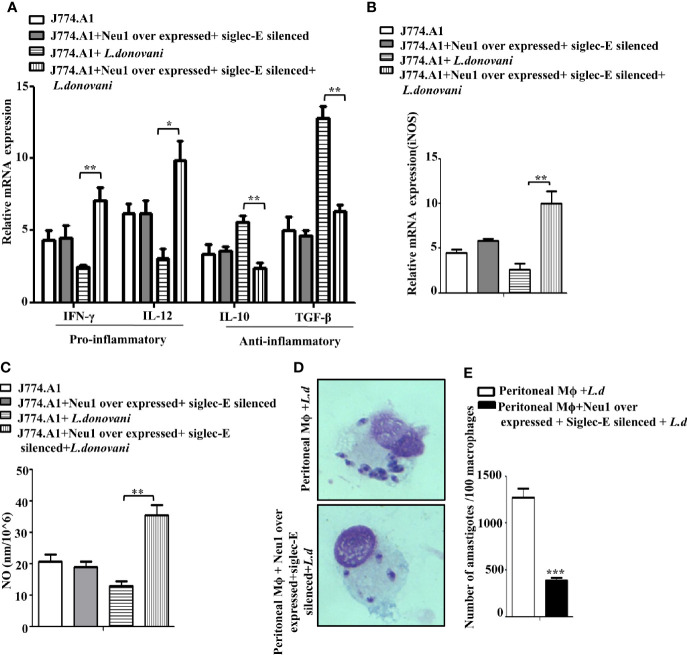
Enhanced immune response in Neu1-overexpressed and siglec-E silenced-co transfected infected cells. J774.A1 cells (1x10^6^/well) were processed as mentioned in **Figure 6** above. **(A, B)** RNA was extracted and relative mRNA expression of these cells was determined using specific primers for **(A)** Pro-inflammatory (IFNγ and IL-12), anti-inflammatory cytokines (IL-10 and TGF-β) and **(B)** iNOS as stated in Materials and Methods. **(C)** Cell-free culture supernatant from these cells was used to determine the secreted nitric oxide level by Griess assay. **(D)** Peritoneal macrophages were isolated from BALB/c mice as described in materials and methods. These cells were co-transfected and infected as mentioned in **Figure 6** above. Thereafter the cells were processed for Giemsa staining. The stained cells were visualized under optical microscopy to determine the parasite burden. **(E)** The internalized amastigotes were quantitated by counting the number of parasites per 100 macrophages. The experiments were repeated thrice and the values were expressed as mean ± SD for three independent experiments. A Student’s t-test was used to evaluate statistical significance; *p ≤ 0.05, **p ≤ 0.01, ***p ≤ 0.001.

Proper immune activation during VL is characterized by an upregulation of IFN-γ and IL-12 and down-regulation of IL-10 and TGF-β. Accordingly, we checked the expression of these immunomodulatory cytokines in Neu1overexpressed and siglec-E silenced co-transfected *L.donovani* -infected conditions (**Figure 7A**). Our data displayed a significantly enhanced expression of pro-inflammatory cytokines, namely IFN-γ (p ≤ 0.0072, ~ 2.9 fold) and IL-12 (p ≤ 0.011 ~ 3.1 fold) in the co-transfected-infected cells compared to the mock-transfected-infected cells. Simultaneously, the cytokines leading to suppression of macrophage function such as- IL-10 exhibited a significantly (p ≤ 0.005) decreased expression by ~2.3 fold and TGF-β displayed a significant (p ≤ 0.0023) reduction by ~ 2.3 fold compared to mock-transfected-infected cells. We also checked the genetic expression of the effector molecule iNOS which was significantly (p ≤ 0.0081) raised by ~5 fold (**Figure 7B**) in the transfected-infected cells compared to mock-transfected-infected cells. This was further supported by significantly (p ≤ 0.0027) enhanced accumulation of nitric oxide by ~3.2fold in the cell supernatant in the co-transfected *L.donovani-*infected macrophages compared to the mock-transfected-infected cells (**Figure 7C**). No such change could be observed in the co-transfected uninfected cells.

Furthermore, a robust immune activation should lead to reduced parasite survival inside the macrophages. We, therefore, checked the status of parasite load inside these transfected macrophages. Accordingly, peritoneal macrophages from BALB/c mice were co-transfected with Neu1-overexpression plasmid and siglec-E siRNA followed by *L.donovani* infection. We observed a reduced number of amastigotes inside these cells compared to mock-transfected-infected cells (**Figure 7D**). This was also corroborated by a significantly reduced (p ≤ 0.001) number of amastigotes per 100 macrophages (~3 fold) compared to mock-transfected-infected cells (**Figure 7E**). These data suggest Neu1 and siglec-E by acting reciprocally could effectively activate the immune response leading to parasite killing. All these data suggest interplay between Neu1 and siglec-E evokes an effective activation of TLR4.

## Discussion

Sialic acids present on cell surface influence immune recognition by altering the conformation of glycoprotein as well as modulating their biological activity. TLR4 is a well-known sialylated cell surface molecule on the macrophage, which induce the activation of both MyD88 and TRIF-dependent pathways with distinct cytokine repertoire (10–12). Although it is known that the parasite *L.donovani* modulates both Myd88 and TRIF-dependent TLR4 activation, the detailed mechanism of such impairment is largely unknown.

Our study conclusively supports the idea that TLR4-activation during *L.donovani* infection is regulated by the interplay of Neu1 and siglec-E depending on who can dominate on the sialic acids of TLR4. We also demonstrated that sialic acid-siglec-axis along with Neu1 play a major role in fine-tuning of immunity and pathogenicity mediated by TLR4 in the *L.donovani*-infected J774.A1 and primary macrophages.

We observed an enhanced association of TLR4 with siglec-E leading to enhanced siglec-E-phosphorylation and activated the ITIM-related inhibitory signaling through the upregulation of both SHP1/SHP2 and their phosphorylation. The addition of exogenous sialidase abolished such binding and enhanced the nuclear translocation of p-65 indicating an activation of the TLR pathway. Also, desialylation of the parasites with neuraminidase pre-treatment reduced SHP1 and SHP2 phosphorylation. The present study further revealed Neu1-overexpression was able to activate the MyD88-dependent pathway but had little effect on the activation of TRIF-pathway.

Furthermore, siglec-E silencing reversed this scenario with an upregulated TRIF-pathway but MyD88-pathway activation remained unchanged. However, simultaneous up and downregulation of Neu1 and siglec-E together activated both MyD88- and TRIF-pathways. The current study thus demonstrated that differential activation of TLR4-pathways during this parasite infection is under the dual regulation of Neu1 and siglec-E through their action on a common sugar namely sialic acids. Depending on the proximity of these sugar molecules with TLR4, it is either activated through desialylation by Neu1 or suppressed when siglec-E binds to the sialic acids on TLR4. Therefore, our study successfully establishes interplay between Neu1 and siglec-E through recognition of TLR4 sialic acids ascertains the mechanism of TLR4-inactivation during *L.donovani* infection thus paving a safe pathway for the survival of this parasite inside the macrophages.

TLRs are the sentinels of the innate immune response that can actively participate in host-pathogen interaction. However, enhanced sialic acids on TLR4 during *L.donovani* infection due to reduced Neu1 on membrane leads to a defect in TLR4 activation (15). Siglec-E-TLR4 association through cis interaction leads to its phosphorylation and activation of inhibitory-signaling (21, 37, 38). This restricts over-activation of immune cells possibly through TLR4 (25, 39). Additionally, hypersialylated tumor cells engaged siglec-9/siglec-E to modulate the host’s immune response by recognizing such enhanced sialic acids to activate the inhibitory ITIM-signaling (40). Siglecs can also recognise sialic acids on adjacent cells through trans interaction which also leads to inhibition of signaling. Overexpression of siglec-9 in LPS-stimulated THP1 cells reduced proinflammatory cytokines (41) and IL-12 production in human monocyte-derived dendritic cells (42). Murine siglec-E also downregulate TLR4-driven cytokine production in bone marrow-derived macrophages with anti-siglec-E antibody-treatment followed by LPS-stimulation (39). Additionally, direct interaction of siglec-E with TLR4 in THP1 cells slowed down the activity of the receptor (21).

During *L.donovani* infection, SHP1 is upregulated (43), nitric oxide production is downregulated (44) and reprogrammed TLR4-activation (45). Furthermore, SHP1 was exploited by Leishmania to inactivate IL-1 receptor-associated kinase 1 (IRAK-1) and thereby subvert the TLR-signaling (46). Here, we also observed an enhanced siglec-E-TLR4 association during *L.donovani* infection, activation of the inhibitory signaling of siglec-E by up-regulating SHP1/SHP2 as well as their phosphorylation thus corroborating with the previous reports. Such enhanced TLR4-siglec-E association was reduced when host sialic acids were blocked with lectin MALII that binds to α2,3-linked sialic acids. Presence of α2,3-linked sialic acids has been reported on TLR4; removal of which is responsible for its activation (20). Therefore, TLR4-siglec-E interaction was also reduced and the nuclear translocation of NF-κB was elevated when exogenous neuraminidase was added. All these indicated the involvement of sialic acids in such interaction.

Additionally, the interaction of siglec-E with sialic acids on *L.donovani* subverted the immune activation in infected macrophages (30). Our data suggest when parasites was treated with neuraminidase they possibly could not induce hypersialylation and subsequently decreased TLR4-siglec-E interaction preventing the inhibitory ITIM signaling. Moreover, a reduced level of Neu1 on the *L.donovani-*infected macrophages impaired TLR4-signaling. This condition was reversed upon Neu1 overexpression (15). Therefore, activation of TLR4 during *L.donovani*-infection may depend on two regulatory molecules (Neu1 and siglec-E) both of which identify the sialic acids on TLR4 as their target.

To further confirm this dual regulation of TLR4 activation by Neu1/siglec-E, we separately overexpressed Neu1 and silenced siglec-E followed by infection. Overexpressed Neu1 was able to effectively desialylate TLR4 which in turn prevented its association with siglec-E. Conversely, silencing siglec-E inhibited its binding to sialic acids on TLR4. Under such conditions, the association of SHP1/SHP2 with siglec-E was also reduced. This probably indicates the siglec-E-TLR4 association *via* sialic acids during parasite infection is essential to initiate the ITIM-mediated signaling.

It was shown in LPS treated dendritic cells, the siglec-E-TLR4 association was reduced due to endogenous Neu1 (21). Similarly, the interaction of siglec-E with SHP1/SHP2 following phosphorylation at tyrosine residue was reversed in siglec-E knockdown condition in LPS-stimulated macrophages (39). Although our present data showed an association of SHP2 with siglec-E was abolished in siglec-E-silenced condition, interestingly an interaction was still observed when Neu1 was overexpressed. Silencing siglec-E, therefore, completely inhibits any association with sialic acids either with host or the parasite and prevents inhibitory signaling.

We have demonstrated earlier that *L.donovani* cell surface is highly decorated with α2,3- and α2,6-linked sialic acids (47), which in turn leads to its enhanced binding with siglec-E and downregulates the immune response (30). Moreover, siglec-E can bind to both α2,3- and α2,6-linked sialic acids with equal affinity (48). TLR4 on host cell surface predominantly has α2,3-linked sialic acids (20). Therefore, siglec-E can either bind to sialic acids of TLR4 on host cells or it can bind with parasite sialic acids or to both simultaneously. Therefore, under Neu1 overexpressed condition siglec-E still persists in the cell which might interact with the sialic acids on the parasite in trans to sustain its association with SHP2. Siglec-E can interact with sialic acids on host *via* cis and with those on the parasite by trans interaction. Sialic acid binding with siglec-E leads to its signaling activation by enhanced recruitment of SHP1 and SHP2 along with their upregulated phosphorylation. During infection TLR4 has been reported to be hypersialylated (15) which probably serve as the ligands for siglec-E and subsequently initiates inhibitory signaling. Also sialic acids on the parasite interact with siglec-E to initiate immune inhibition. Therefore, possibly siglec-E associates with both host and parasite sialic acid to activate its signaling. We have demonstrated removal of sialic acids from host or parasite diminishes siglec-E-TLR4 interaction. Overexpression of Neu1 followed by infection was able to prevent sialic acid siglec-E interaction through TLR4 desialylation. However, presence of siglec-E in the Neu1 overexpressed condition was able to maintain its association with sialic acids on the parasite in trans that possibly led to enhanced SHP2-siglec-E association even under Neu1 overexpression. This in turn prevents the TRIF mediated TLR4 signaling. Thus *L.donovani* through its sialic acids also plays a role in mediating a cross talk among TLR4, Siglec E and Neu1.

Furthermore, considering the numerous intracellular signaling networks and the complex signal crosstalk, other signals may also determine which SHP would be recruited by siglec-E and what SHP will take the upper hand. Therefore, both sialic acids on the host cell as well as the parasite play an important role in the siglec-E-TLR4 interaction and the outcome of immune activation during this disease.

Sialic acids on TLR4 are recognized by both Neu1 and siglec-E during *L.donovani* infection. When Neu1 alone was overexpressed followed by infection, it led to the activation of the MyD88-pathway possibly due to TLR4-desialylation (20). Moreover, as the association of TLR4 with MyD88 was slightly upregulated in the siglec-E-silenced-infected cells, it may be envisaged that Neu1 plays a dominant role in the activation of MyD88 pathway. But such Neu1-overexpression failed to activate the TRIF-pathway may be due to a strong association of SHP2 with siglec-E. A similar condition leading to SHP2-mediated subversion of IFN-β; a downstream gene of TRIF-pathway was reported in RNA virus-infected macrophages (49). Furthermore, earlier reports suggest SHP2 negatively regulates TLR4 activation through inhibition of TRIF (50). Siglec-E has also been demonstrated to downregulate TRIF in LPS-stimulated macrophages (39). Our data corroborated with such observation where, in contrast to Neu1-overexpression, silencing-siglec-E alone specifically activated the TRIF-pathway which was suppressed in the untransfected-infected cells. Under the siglec-E-silenced-infected condition, Neu1 was still down modulated in those cells, consequently, TLR4 was hypersialylated. This, in turn, prevented MyD88-dependent TLR4-activation. Furthermore, the removal of sialic acids on TLR4 is crucial for Myd88-mediated TLR4-signaling (20). Therefore, reduced Neu1, as well as enhanced siglec-E-TLR4 association through interaction with sialic acids, down-regulated both MyD88- and TRIF-pathways during infection. It has been documented in MyD88^-/-^ bone marrow macrophages infected with *L.panmensis*, TNF-α production was significantly downregulated. The absence of TRIF, however, did not alter TNF-α under such conditions (51). Furthermore, Nagala et al. have reported silencing siglec-E in macrophages followed by *E.coli* infection failed to alter TNF-α and IL-6 inflammatory cytokine production (52). Our present data matched with such observation where MyD88-activation upon Neu1-overexpression enhanced TNF-α and Il-6 production but the absence of siglec-E did not, indicating activation of these cytokines is specific to MyD88-activation. On the contrary, siglec-E expression was shown to be responsible for inhibiting TRIF-driven IFN-β in LPS-treated macrophages (39). Silencing siglec-E reversed such observation. Thus, a reciprocal relationship between Neu1 and siglec-E is responsible for the impairment of TLR4 signaling.

The ubiquitination of proteins plays a crucial role in immune regulation. Furthermore Triad3A, an E3 ubiquitin ligase, causes ubiquitin-dependent TLR4-degradation in *E.coli*-infected macrophages (25, 53). This intrigued us to investigate if modulation of Neu1 and siglec-E has a protective role in such degradation during infection. While more ubiquitination of TLR4 followed by its strong association with Triad3A was observed in untransfected-infected cells, such association was reduced in the Neu1-overexpressed or siglec-E-silenced infected cells. During infection, either reduced Neu1 or enhanced siglec-E-TLR4 association ubiquitinates and degrades TLR4. Such degradation was reduced when this condition was reversed as more TLR4 was present on the cell membrane. Therefore, these two players probably maintain TLR4 levels by modulating the ubiquitination and thus activation of the immune response.

However, overexpressing Neu1 and silencing siglec-E together followed by infection was able to effectively upregulate both MyD88- and TRIF-dependent pathways of TLR4 activation. Under the above condition, more Neu1 associated with TLR4 leading to its desialylation and MyD88-signaling activation which upregulated its corresponding cytokines. Simultaneously, siglec-E silencing decreased its binding with TLR4 and also prevented it from interacting with sialic acids on the parasite. This ultimately removed the inhibitory effect of siglec-E leading to TRIF-dependent IRF3-activation. All these led to up-regulation of macrophage activating pro-inflammatory and down regulation of anti-inflammatory cytokines and thus reduced parasite survival inside the host cells.

**Figure 8:**
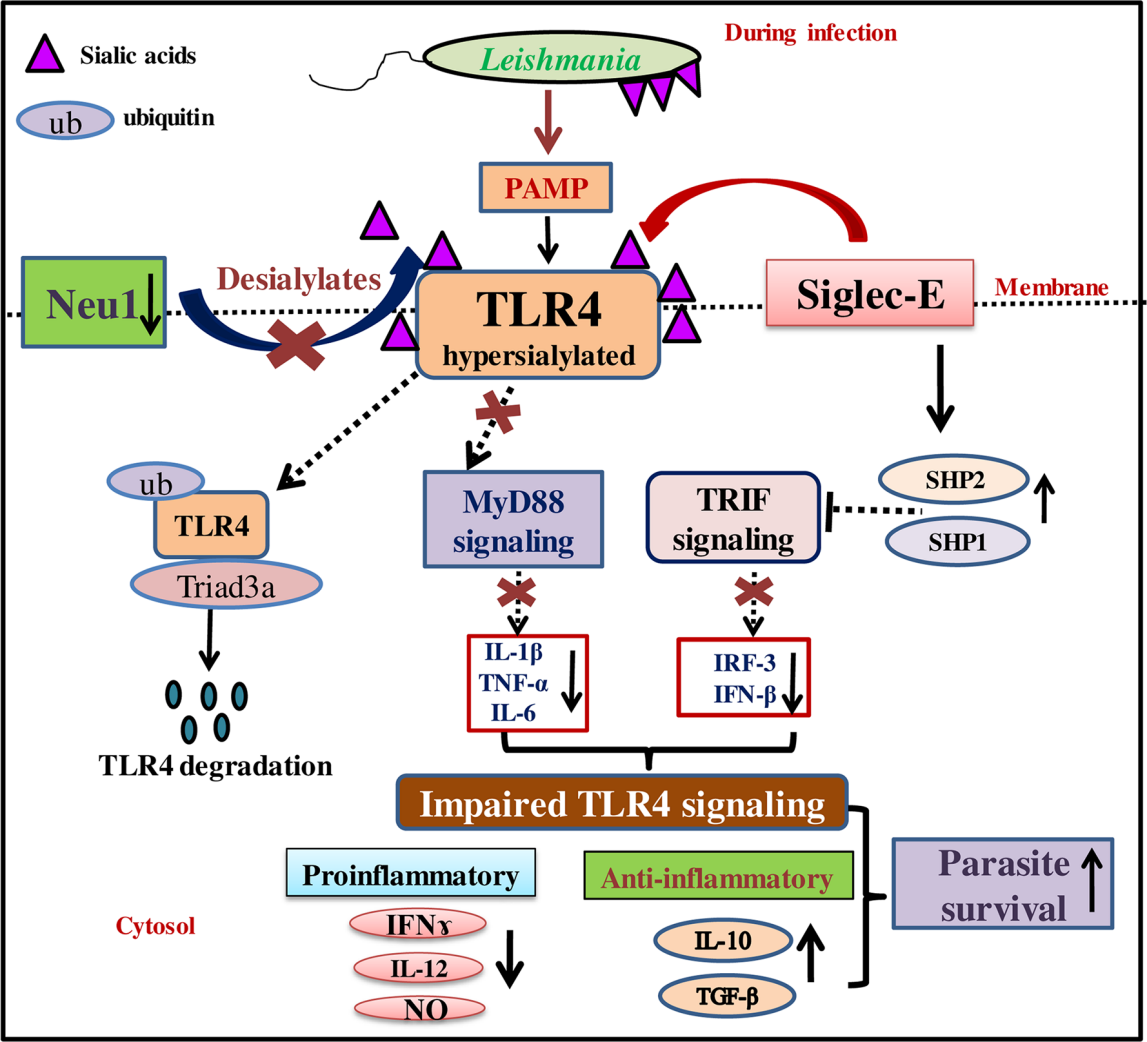
Schematic representation demonstrating modulation of MyD88- and TRIF-dependent toll-like receptor 4 (TLR4)-activation by interplay between Neu1 and siglec-E during *L.donovani* infection. Reduced Neu1 during *L.donovani* infection hypersialylates toll-like receptors (TLR4) (15) preventing MyD88-mediated TLR4 activation. Simultaneously, siglec-E binds hypersialylated TLR4 to activate inhibitory signaling through activation of SHP1 and SHP2. This leads to ubiquitin-mediated TLR4 degradation by Triad3A. All these events jointly dysregulate the cytokine repertoire of MyD88- and TRIF-pathways. This, in turn, supports parasite survival through the up-regulation of Th2 cytokines and down-regulation of Th1 cytokine along with decreased effector molecule nitric oxide. Thus sialic acids on TLR4 play a crucial role in its activation through interplay between Neu1 and siglec-E.

Taken together, our present study demonstrates a fine balance between activation and inhibitory signal through Neu1 and siglec-E is crucial for effective host defense mediated by TLR4. Altogether, the data indicate activation of MyD88- and TRIF-pathways depends on whether Neu1/siglec-E binds to sialic acids on TLR4 thus confirming their interplay which is responsible for an impaired TLR4-signaling during *L.donovani* infection (**Figure 8**). Such understanding of the involvement of Neu1/siglec-E in TLR4-activation may allow the exploitation of these molecules in the development of immunotherapy during VL. As the sialidase and siglec-sialic acid axis play an important role in maintaining a balance between immunity and tolerance, future studies are of utmost importance to understand their role in resolving disease pathogenesis.

## Data Availability Statement

The original contributions presented in the study are included in the article. Further inquiries can be directed to the corresponding author.

## Ethics Statement

The animal study was reviewed and approved by Institutional Animal Ethics Committee (IAEC) of CSIR-Indian Institute of Chemical Biology (IICB), Kolkata, India.

## Author Contributions

JK conceived the work, performed all the experiments and analyzed the data. CM supervised the whole work. Both authors contributed to the article and approved the submitted version.

## Funding

The work was supported in part by CSIR-HCP010; DBT-GAP346; SERB, GAP 336/GAP 339 and Indian Council of Medical Research, GAP 370.

## Conflict of Interest

The authors declare that the research was conducted in the absence of any commercial or financial relationships that could be construed as a potential conflict of interest.
